# Supplementary material for: The Effect of Cardiopulmonary Resuscitation (CPR) Education on the CPR Knowledge, Attitudes, Self-Efficacy, and Confidence in Performing CPR among Elementary School Students in Korea
Source: Healthcare (Basel). 2023 Jul 17;11(14):2047. doi: 10.3390/healthcare11142047 (PMC10379098; doi:10.3390/healthcare11142047)
Supplement: Supplementary file 1 [file healthcare-11-02047-s001.zip › healthcare-2496667-supplementary.pdf]

Supplementary Materials

Table S1. CPR Knowledge.

Questions	Before CPR education N(%)	After CPR education N(%)	Pearson chi-square	p-value
1. If you saw a person collapse suddenly, which of the following is the first thing to do?	81(57.9)	98(70.0)	4.476	0.034*
2. What is the appropriate way to determine a patient's state of consciousness?	68(48.6)	120(85.7)	43.774	0.000***
3. What is the right way to check the breathing of a patient who suddenly collapsed?	13(9.3)	19(13.6)	1.270	0.260
4. How many chest compressions should you perform per minute?	87(62.1)	105(75.0)	5.369	0.020*
5. How do you do artificial respiration?	100(71.4)	93(66.4)	0.817	0.366
6. What is the proper position of the hand when performing chest compression?	130(92.9)	133(95.0)	0.567	0.453
7. If an unconscious person is an adult, what is the correct position to check the pulse?	56(40.0)	57(40.7)	0.015	0.903
8. After cardiac arrest, when does brain damage begin without CPR?	85(60.7)	95(67.9)	1.556	0.212

* ($p < 0.05$), *** ($p < 0.001$).

Table S2. Attitude toward CPR.

Question	Before CPR education (M±SD)	After CPR education (M±SD)	t	p-value
1. I think that a non-emergency paramedic or non-medical person can perform CPR.	3.77±1.127	3.98±1.184	1.499	0.135
2. I think that performing CPR increases the possibility of survival.	3.75±0.945	4.10±0.884	3.200	0.002**
3. I think that when a collapsed person is unconscious, performing CPR is necessary.	3.41±1.045	3.76±0.895	3.072	0.002**
4. I think that CPR should be provided, even if there is any chance of getting sick from the patient.	2.89±1.093	3.45±0.947	4.616	0.000***
5. I am usually interested in CPR and try to learn CPR.	2.84±1.242	3.06±1.037	1.567	0.118
6. I think I need to learn CPR.	4.21±1.103	4.16±0.939	0.409	0.683
7. I think it is good to have frequent opportunities for CPR education.	3.97±1.079	3.74±1.013	1.827	0.069
8. I am willing to participate in CPR education again.	3.75±1.236	3.70±1.044	0.366	0.715
9. If I receive CPR education, I am willing to perform CPR in emergent situation.	3.35±1.099	3.64±0.945	2.391	0.017*

10. I think that CPR education should be provided in schools.	3.77±1.140	3.86±0.946	0.742	0.459
11. I am willing to perform mouth-to-mouth ventilation.	2.61±1.160	2.78±1.039	1.248	0.213

* ($p < 0.05$), ** ($p < 0.01$), *** ($p < 0.001$).

Table S3. Self-efficacy for CPR.

Question	Before CPR education (M±SD)	After CPR education (M±SD)	t	p
1. I can check the patient's abnormal breathing or apnea.	3.39±1.043	3.28±0.997	0.843	0.400
2. I can judge the timing of the request for help.	3.38±1.255	3.68±0.884	2.313	0.021*
3. If I find a cardiac arrest patient, I can ask for help.	4.12±1.007	4.08±0.960	0.364	0.716
4. I can check pulse in the carotid artery of an unconscious patient.	2.64±1.151	3.03±1.092	2.876	0.004**
5. I can perform CPR for a cardiac arrest patient.	3.04±1.141	3.54±0.970	4.008	0.000***
6. I can evaluate the cardiac arrest situations.	2.76±1.185	3.16±0.845	3.252	0.001**
7. I can check if a patient is conscious or unconscious.	4.07±1.043	4.00±0.996	0.586	0.558
8. I can call 119 when the patient is unconscious.	4.52±0.791	4.26±0.934	2.486	0.013*
9. I can perform the mouth-to-mouth ventilation.	2.79±1.128	3.02±1.160	1.671	0.096
10. I can point out the correct hands position for chest compression.	3.20±1.242	3.66±1.009	3.380	0.001**
11. I can perform chest compression with the correct depth of chest compression.	2.96±1.166	3.44±1.088	3.551	0.000***
12. I can perform chest compression with the correct rate of chest compression.	2.95±1.189	3.47±1.109	3.794	0.000***
13. I can turn on the AED.	3.16±1.355	3.14±1.083	0.146	0.884
14. I can attach the pads of the AED in the correct position.	2.91±1.275	2.97±1.125	0.447	0.655
15. I can use the AED.	2.90±1.219	2.91±1.099	0.051	0.959
16. I can perform CPR in the correct order according to the algorithm.	2.99±1.252	3.21±0.988	1.696	0.091
17. I can explain what I did well and what I did poorly after performing CPR.	2.97±1.223	3.28±0.997	2.303	0.022*

* ($p < 0.05$), ** ($p < 0.01$), *** ($p < 0.001$).
